# Genome-Wide Identification of the *bHLH* Gene Family in *Magnolia sieboldii* and Response of *MsPIFs* to Different Light Qualities

**DOI:** 10.3390/ijms26073152

**Published:** 2025-03-28

**Authors:** Lin Liu, Xin Xu, Xiaohuan Yang, Hanzhang Liu, Lingyi Xu, Wanfeng Ai, Xiujun Lu

**Affiliations:** 1 College of Forestry, Shenyang Agricultural University, Shenyang 110866, Chinawfai23@syau.edu.cn (W.A.); 2 Key Laboratory for Silviculture of Liaoning Province, Shenyang 110866, China

**Keywords:** *Magnolia sieboldii*, *bHLH* gene family, seed germination, different light quality

## Abstract

The basic helix-loop-helix (*bHLH*) gene family has been identified in many species. However, the characteristics and functions in the *Magnolia sieboldii* K. Koch (*M. sieboldii*), which is located in one of the original groups of angiosperms, are still unclear. Here, a total of 142 *MsbHLH* members were identified and divided into 27 subfamilies. MsbHLH proteins are relatively conserved during evolution. Collinearity analysis illustrated that the expansion of the *MsbHLH* gene family primarily occurred through segmental duplication. All members contain light-responsive elements in their promoters. Different light quality treatments were carried out to simulate the light environment in the forest after seed abscission. It was found that the expression levels of *MsPIF1*, *MsPIF3b*, *MsPIF4*, and *MsPIF7* gradually increased under far-red light and inhibited seed germination. Overall, this study lays the foundation for further exploration of the response mechanism of *MsPIFs* to seed germination under different light qualities. It will provide a reference for the germination of morphophysiological dormant seeds like those of *M. sieboldii* under light conditions.

## 1. Introduction

*Magnolia sieboldii* K. Koch (*M. sieboldii*) is a deciduous small arbor belonging to the Magnoliaceae family and the Magnolia genus, with a discontinuous geographical distribution. The class of Magnoliids to which it belongs has been controversial in recent years in the systematic classification of angiosperms. Whether it is at the base of the true dicotyledons or throughout the monocotyledons has not been decided. Therefore, *M. sieboldii* holds an important classification status and belongs to a more primitive group of angiosperms [1]. There is a need for its study. It has the value of ornamental, aromatic oil extraction, and medicinal use, and is a rare wild woody plant resource with significant potential for development and utilization [2]. Research shows that the seeds of *M. sieboldii* are typical seeds with morphological and physiological dormancy [3]. There are a number of causes of seed dormancy in Magnolia sieboldii, including incomplete development of the seed embryo, the presence of germination inhibitors [4], and the high concentrations of ABA and IAA, as well as low concentrations of GA_3_ in the endosperm at the time of seed maturity [5]. Therefore, it is significant to explore the mechanisms regulating the release of dormancy and germination in *M. sieboldii* seeds for the propagation of such seeds with morphological and physiological dormancy characteristics and for further plant conservation.

The basic helix-loop-helix (bHLHs) proteins are a class of transcription factors found throughout eukaryotes, initially discovered in animals [6]. The bHLH domain consists of 50–60 conserved amino acid (aa) residues. These include 10–15 DNA-binding segments of predominantly basic amino acids, followed by a segment of approximately 40 amino acids that includes two α-helices separated by a variable loop region (HLH) [7]. The HLH region facilitates dimerization, allowing for the formation of homodimer or heterodimer complexes among different family members [8,9].

The bHLH motifs seem to have been established early in the evolution of eukaryotes. In yeast, *bHLH* genes are involved in general transcription enhancement and cell cycle regulation, suggesting that these may have been the original functions of *bHLH* genes in primitive eukaryotes [10]. Research indicates that the diversity of bHLH proteins in angiosperms is ancient. Most bHLH subfamilies are found in seed plants, such as Arabidopsis and rice. As well as in early-diverging terrestrial plant groups, including mosses and lycophytes. In contrast, the diversity of bHLH proteins is significantly lower in green algae and red algae. This suggests that the bHLH family underwent a major expansion shortly before or after the emergence of the first terrestrial plants. However, it subsequently remained relatively conserved throughout the evolutionary process of terrestrial plants [11].

bHLH transcription factors play a significant role in various growth and developmental processes in plants, including seed germination, flowering time regulation, epidermal cells, stomata, and fruit maturation [12,13]. The dormancy and germination of seeds are influenced by external environmental factors such as light, temperature, and humidity, as well as internal factors. Phytochrome interacting factors (PIFs) are a group of bHLH proteins. One of them, PIF1 (PIL5), is an inhibitor of seed germination and acts by reducing GA levels in the dark. Phytochromes facilitate the degradation of PIF1, thereby increasing the bioactive levels of GA in seeds to promote germination [14]. Another bHLH transcription factor, SPT, also influences seed germination through its interaction with PIF1 [12,15]. Additionally, regulating flowering time in plants is essential. Four bHLH-related proteins, FBH1, FBH2, FBH3, and FBH4, act as transcriptional activators of the *CO* (commanding officer) gene. These proteins bind to the E-box *cis*-acting elements in the CO promoter, positively regulating flowering time through the action of CO [16]. In addition, many bHLHs also play crucial roles in responses to abiotic stress [17]. In rice, the overexpression of *OsbHLH148* can regulate the jasmonic acid pathway and the function of jasmonic acid ZIM domain proteins, thereby enhancing the plant’s drought resistance [18,19]. In tomato, overexpression of *SlbHLH22* improves salt tolerance, enhances ROS scavenging, and increases osmoregulatory potential in tomato seedlings [20]. Plants often require bHLH transcriptional activity to regulate various physiological and biochemical processes in response to low-temperature stress [21]. Systematic genomic analysis has preliminarily indicated that several *bHLH* genes in sweet cherries (*PavbHLH1*, *PavbHLH18*, *PavbHLH28*, *PavbHLH60*, *PavbHLH61*, *PavbHLH65*, and *PavbHLH66*) are involved in the response to cold stress [22]. The *DlICE1* gene derived from longan can enhance the cold resistance of transgenic Arabidopsis by increasing proline content in seedlings and reducing malondialdehyde levels [23].

Systematic identification and research on *bHLH* genes across various species can enhance our understanding of their evolution and function. However, there are limited reports on this topic in more primitive plant groups during the evolution of angiosperms. This study conducted a series of systematic analyses, including chromosomal localization, collinearity analysis, evolutionary relationships, gene structure, and motif composition, on 142 identified *MsbHLH* genes. It comprehensively explored the *bHLH* gene family in *M. sieboldii* and its expression during the seed germination process, thereby laying a solid foundation for future functional studies on seed dormancy release and germination.

## 2. Results

### 2.1. Whole-Genome Identification of bHLH TFs in M. sieboldii

**Figure 1:**
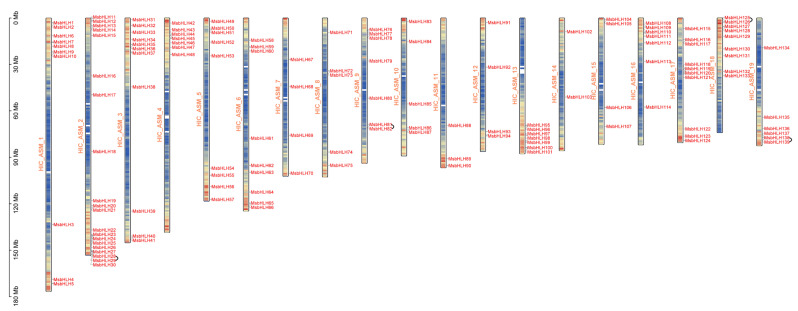
Distribution of *MsbHLH* genes in chromosomes and tandem repeat events. Chromosome names were located on the left side of the chromosomes, indicated by HIC_ASM_. The position of each *MsbHLH* was recorded on the right side of the corresponding chromosome. Chromosome size was indicated by its relative length. Tandem duplicated genes were indicated by black arcs connecting them.

In this study, we identified a total of 142 gene family members (Table S1). They were renamed *MsbHLH1*-*MsbHLH142*, where *MsbHLH140*-*MsbHLH142* could not be localized to any chromosomal scaffold. The remaining 139 MsbHLH members were unevenly distributed in the 19 chromosomes (Figure 1), with chromosome 2 (HIC_ASM_2) containing the maximum number of genes with 20 genes (*MsbHLH11*-*MsbHLH30*), followed by chromosome 3 (HIC_ASM_3) with 11 genes (*MsbHLH31*-*MsbHLH41*). Chromosome 14 (HIC_ASM_14) had the minimum number of genes, with only 2 *MsbHLH* genes (*MsbHLH102* and *MsbHLH103*).

**Figure 2:**
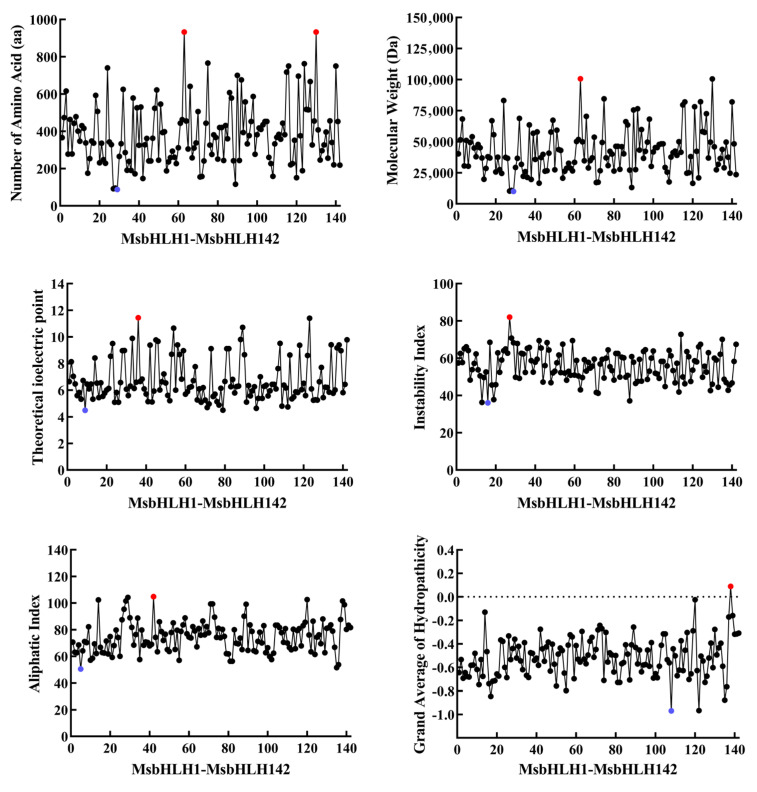
Physicochemical properties of 142 MsbHLH proteins. Maximum values were indicated by red dots and minimum values by blue dots in each figure.

The physicochemical properties of proteins play a crucial role in the biological function and conformation of proteins [24], which are the most fundamental properties of proteins and the first thing that needs to be determined for the research and analysis of proteins. Because of the differences in the molecular weight, isoelectric point, and other properties of each amino acid, the physicochemical properties of proteins made from their combination will also show different characteristics. We analyzed the physicochemical properties of these 142 MsbHLH proteins (Figure 2, Table S2), and the proteins encoded by the 142 *MsbHLHs* contained a range of 88 (*MsbHLH29*) to 933 (*MsbHLH63*, *MsbHLH130*) amino acids, with molecular weights ranging between 10,025.56 Da (*MsbHLH29*) and 100,730.7 Da (*MsbHLH63*). The predicted theoretical isoelectric points of the proteins ranged from 4.48 (*MsbHLH9*) to 11.44 (*MsbHLH36*). The instability index is used as a reference value for the stability of the protein in vitro tests. Below 40 indicates good stability, above 40, the protein may be unstable. The instability index means that the protein may be more susceptible to degradation or misfolding within the cell, thus affecting its function. Except for MsbHLH13, MsbHLH16, MsbHLH19, and MsbHLH88 proteins, the instability index of all the proteins was greater than 40, indicating that the rest of MsbHLH proteins were unstable in vitro. The aliphatic index can be a favorable factor for the increased thermal stability of globular proteins. The results of aliphatic index prediction showed that the aliphatic index of MsbHLH proteins ranged from 50.5 (MsbHLH5) to 104.83 (MsbHLH42). The hydrophilicity of amino acids is one of the main drivers of protein folding, so the distribution of protein hydrophilicity can respond to protein folding. All the proteins had negative values except for MsbHLH138, which had an overall mean hydrophilicity score (GRAVY) of positive 0.09, indicating that only MsbHLH138 is hydrophobic and the rest are hydrophilic proteins. Subcellular localization predictions showed that a total of 126 MsbHLH proteins were localized in the nucleus, with 2 MsbHLHs localized in the plasma membrane (MsbHLH24, MsbHLH92), 4 MsbHLHs localized in the extracellular space (MsbHLH15, MsbHLH40, MsbHLH43, MsbHLH67), and 9 MsbHLHs localized in the chloroplast (MsbHLH36, MsbHLH42, MsbHLH71, MsbHLH89, MsbHLH103, MsbHLH113, MsbHLH137, MsbHLH138, MsbHLH139), and 1 MsbHLH localized to mitochondrion (MsbHLH75).

### 2.2. Phylogenetic Relationship Between AtbHLHs and MsbHLHs

**Figure 3:**
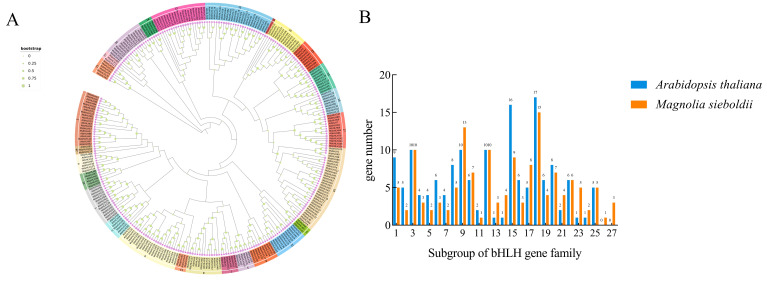
Phylogenetic analysis of MsbHLHs and AtbHLHs: (**A**) The phylogenetic tree includes 142 MsbHLH proteins as well as 153 AtbHLH proteins. Purple circles represent MsbHLH, and purple pentagrams represent AtbHLH. Green circles represent bootstrap, ranging from 0 to 1 from small to large, respectively. (**B**) Number of MsbHLH and AtbHLH in each subfamily.

In order to explore the potential biological relationship between *MsbHLHs* and *AtbHLHs*, an evolutionary tree was constructed using the Neighbor-Joining (NJ) method (Figure 3). According to the taxonomic relationships of *AtbHLHs* in previous studies [25,26], *MsbHLHs* are classified into 27 subfamilies, exhibiting a non-uniform distribution among the subfamilies. The number of members in each subfamily ranges from 1 to 15, with subfamily 18 containing the largest number of *MsbHLH* members at 15. Subsequently, subfamily 9 contains 13 *MsbHLHs*. Conversely, subfamilies 11 and 26 exhibit the fewest number of members, with only 1 member (*MsbHLH87* and *MsbHLH119*), respectively.

### 2.3. Gene Structure, Motif, and Intron–Exon Analysis

**Figure 4:**
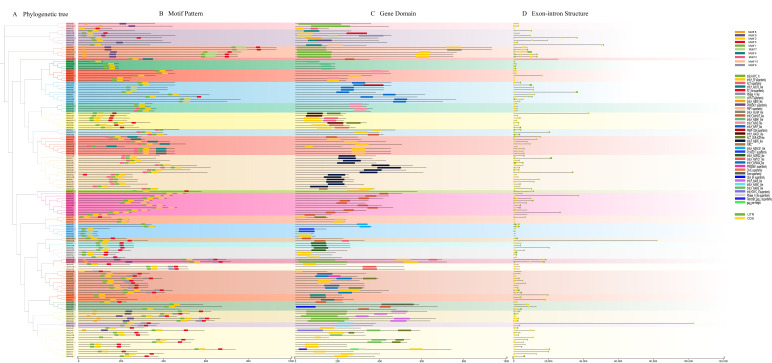
Phylogenetic relationships, conserved motifs, contained structural domains, and gene structures of *MsbHLHs*: (**A**) Phylogenetic tree of MsbHLH proteins. (**B**) Distribution of conserved motifs in MsbHLH proteins. A total of 10 motifs were detected and the scale bar indicates 200 aa. (**C**) Distribution of structural domains in *MsbHLHs.* (**D**) Gene structures of *MsbHLHs*.

A conserved motif search for 142 MsbHLH proteins was conducted through the online site MEME, with the objective of analyzing their gene structures and conserved motif compositions (Figure 4B) to explore the diversity of motifs in MsbHLH proteins. Different MsbHLH proteins contain different numbers of motifs, ranging from 1 to 6. The overwhelming majority of MsbHLH proteins contain motif 1 and motif 2, with these two motifs situated in close proximity to one another. This observation suggests that these two motifs may be associated with the bHLH structural domain. All members of subfamily 23 contain only motif 1, but not motif 2. Typically, members of the same subfamily also have highly similar motif positions and compositions. For instance, all five members of subfamily 23 MsbHLH proteins contain motif 1, motif 5, motif 7, motif 8, and motif 9. This evidence also indicates that different MsbHLH proteins are relatively conserved within the same subfamily and may have similar functions. However, certain motifs are only present in specific subfamilies. For example, motif 7 is only present in subfamily 23, while motif 10 is only observed in subfamily 12. This suggests that these two motifs may have specific functions in these two groups.

Meanwhile, we analyzed the structural domains containing 142 *MsbHLHs* (Figure 4C), and there were 36 structural domains. Of these, 19 types of bHLH-conserved structural domains were identified. Members of the same subfamily, except for some individual genes, have similar structural domain types.

In order to gain further insight into the evolution of the *bHLH* gene family in *M. sieboldii*, we performed intron–exon prediction (Figure 4D). The results demonstrated that members of the same subfamily exhibited greater similarity in gene structure, indicating a strong correlation between phylogenetic evolutionary relationships and gene structure. The number of exons in the 142 *MsbHLHs* varied from 1 to 12. Among them, the number of *MsbHLHs* containing 4 CDSs and 7 CDSs is as high as 21, while the number of *MsbHLHs* containing 12 CDSs is the lowest, only *MsbHLH118*. *MsbHLH25* has five UTRs, which suggests that its differential splicing form is more complex.

### 2.4. Intraspecific and Interspecific Collinearity and Gene Duplication Analysis

**Figure 5:**
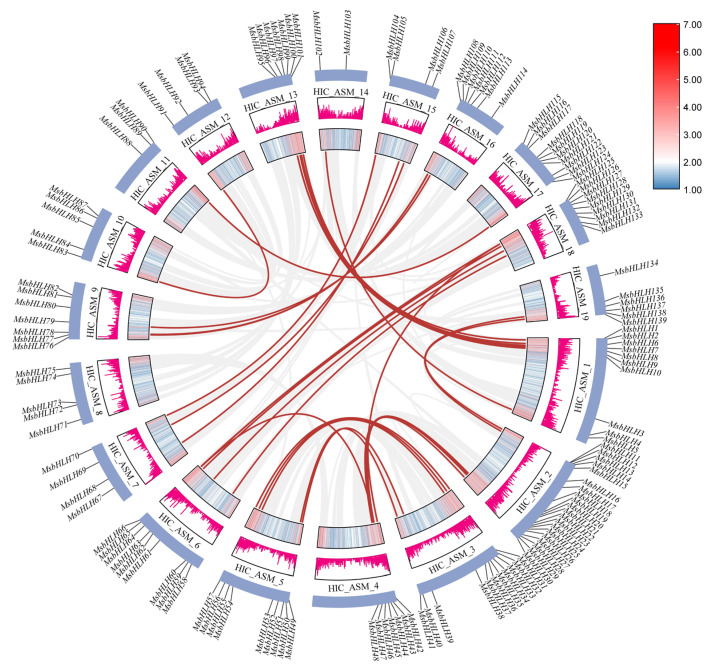
Distribution and collinearity of *MsbHLHs* in the *M. sieboldii* chromosomes. The red lines represent the collinearity between *MsbHLH* genes. The blue boxes represent the *M. sieboldii* chromosomes. The two boxes inside each chromosome represent its gene density.

In order to expand the genome, gene duplication often occurs along with the evolutionary process in plants. Genome duplication events include whole genome duplication, segmental duplication, tandem duplication, and translocation duplication [27]. Among the 139 *MsbHLH* genes localized to chromosomes, 4 tandem duplicated gene pairs (Figure 1) (*MsbHLH28*-*MsbHLH29*, *MsbHLH81*-*MsbHLH82*, *MsbHLH125*-*MsbHLH126*, *MsbHLH138*-*MsbHLH139*) and 35 segment duplicated gene pairs (Figure 5) were identified. This included no segmental duplicates on chromosome 8 (HIC_ASM_8), and all *MsbHLH* genes on chromosome 13 (HIC_ASM_13) were colinear with some genes on chromosome 1 (HIC_ASM_1). The aforementioned results indicate that tandem duplication and segmental duplication are the primary forces driving the evolution of the *MsbHLH* gene family.

In genetics, Ka/Ks denotes the ratio between a nonsynonymous substitution Ka and a synonymous substitution Ks, which can determine whether there is selection pressure. Therefore, we calculated the Ka/Ks of 35 segment duplicated gene pairs (Table S3), and the results showed that all of them had Ka/Ks value < 1, indicating that the *MsbHLH* gene pairs may have undergone purifying selection during evolution. The *bHLH* gene sequence similarity is very high, which plays an important role in the retention of the conserved structure of the gene.

**Figure 6:**
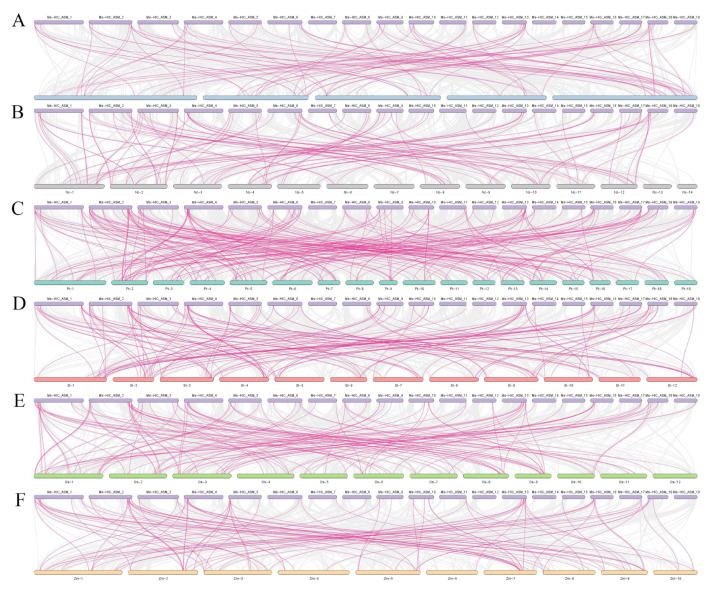
Collinearity of *bHLH* genes between *MsbHLH* and 6 other species. Gray lines represent collinearity between *MsbHLH* and other genomes in each plant. Pink lines represent collinearity between *bHLH* genes. Each box represents the corresponding chromosome: (**A**–**F**) represent collinearity between *MsbHLH* and *Arabidopsis thaliana*, blue-starred waterlily, poplar, tomato, rice, and maize, respectively.

The analysis of collinearity among different species is one way to study their evolution and affinity. To further elucidate the origin and phylogenetic mechanism of the MsbHLH family, we selected four dicotyledonous plants (Arabidopsis, poplar, tomato, and blue star water lily) and two monocotyledonous plants (rice and maize) for analyzing and comparing their homology with *MsbHLHs* (Table S4). A total of 81 *MsbHLHs* were found to be collinearly related to poplar by mapping homology (Figure 6), followed by 77 in blue star water lily, 76 in tomato, 73 in rice, 54 in Arabidopsis, and 53 in maize. Of the 139 *MsbHLHs* localized on chromosomes, 6 *MsbHLHs* were collinearly associated with only 2 monocotyledons and 32 *MsbHLHs* were associated with only 4 dicotyledons. Thus, this suggests that these few members have a closer evolutionary relationship with monocotyledonous or dicotyledonous *bHLHs*. Among all members, there are also 20 *MsbHLHs* that are collinearly related to all 6 plants, suggesting that these genes in the *bHLH* gene family played an important role in the evolutionary process.

### 2.5. Protein Interactions Analysis of MsbHLH Protein

**Figure 7:**
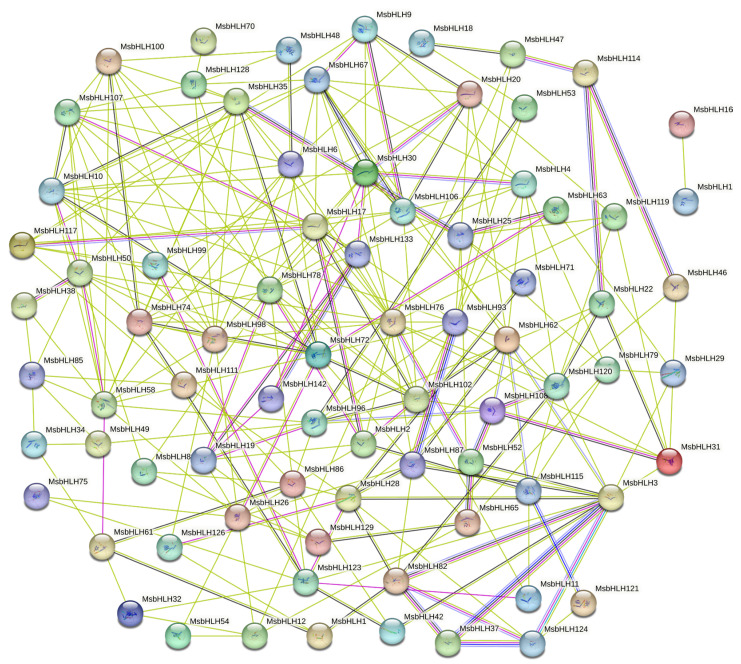
MsbHLH protein interaction network based on Arabidopsis direct homologs. Network nodes represent proteins, and all filled nodes represent proteins whose corresponding 3D structures are known or predicted. Each different colored edge represents the source of its interaction. Light blue indicates from databases. Pink represents determined from experimental data. Green is gene neighborhood. Red is gene fusions. Dark blue is gene co-occurrence. Yellow is text mining. Black is co-expression. Purple is protein homology.

Prediction of protein interaction networks based on homologous proteins of Arabidopsis thaliana by STRING website (Figure 7). It was found that there were 82 nodes with 252 edges in the MsbHLH protein interaction network. Some proteins showed direct interactions with other proteins, such as MsbHLH70 and MsbHLH128. MsbHLH132 and MsbHLH16 only had interactions with each other and did not interact with other MsbHLH proteins. Others showed more complex interactions, which were radially connected to other nodes, such as MsbHLH93, MsbHLH3, MsbHLH78, etc., illustrating the diverse functions of MsbHLH proteins. Table S5 demonstrates the detailed information of MsbHLH proteins compared with AtbHLH proteins.

The results of protein secondary structure prediction (Table S6) showed that MsbHLH proteins were mainly composed of α-helices, extended strands, β-turns, and random coils. α-helices range from 17.82% (MsbHLH124) to 70.45% (MsbHLH29); extended strands range from 0.00% (MsbHLH29, MsbHLH36, MsbHLH54) to 20.18% (MsbHLH137); β-turns range from 0.00% (MsbHLH28, MsbHLH29) to 8.11% (MsbHLH137); and random coils range from 29.03% (MsbHLH71) to 75.29% (MsbHLH39). Protein secondary structure changes have a wide range of effects on the sequence, structure, stability, abundance, and activity of proteins [28].

Protein tertiary structure is important to fill the gap between protein sequence and protein structure [29]. Based on the SWISS-MODEL database, protein tertiary structure models of 27 subfamilies were predicted, and the ones with the highest GMQE and GMEAN scores were selected as the representative protein tertiary models of each subfamily (Figure S1). The 3D structures of each subfamily were different, and the protein structures were not quite similar even between two subfamilies with close relatives in the evolutionary tree. The analysis of the protein homology models provides initial insights into further understanding of the function of MsbHLH proteins.

### 2.6. Cis-Regulatory Element Analysis of MsbHLHs

**Figure 8:**
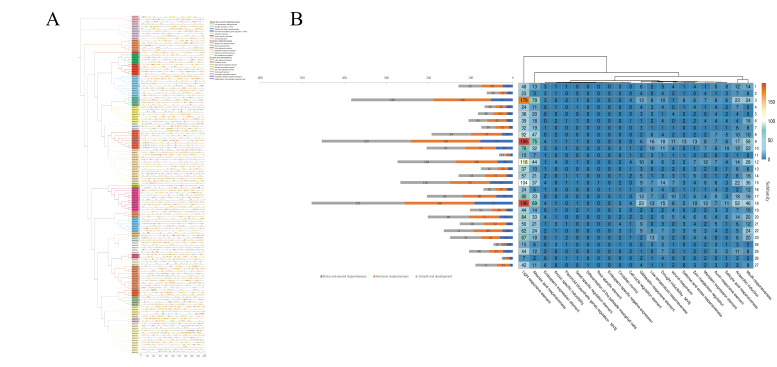
Cis-regulatory elements on the promoters of *MsbHLHs:* (**A**) Distribution of cis-regulatory elements in the 2000 bp upstream promoter region of *MsbHLHs.* (**B**) Number of cis-regulatory elements on the promoter in each subfamily. The distribution of the three major classes of cis-regulatory elements in each subfamily on the left. The distribution of each cis-acting element in each subfamily on the right.

*Cis*-regulatory elements (CREs) are essential for gene expression and regulatory functions. A total of 4296 CREs were detected in 142 *MsbHLH* promoter regions (Figure 8). Subfamily 18 of *MsbHLH11* had the maximum number of CREs, totaling 67. Subfamily 17 of *MsbHLH70* had the minimum number of CREs, only 12. CREs varied with subfamily, but in total, they could be divided into three main categories, namely, elements related to stress response, elements related to hormone response, and elements related to growth and development. The maximum number of CREs related to growth and development, totaling 2014. Among these, there were 1796 light-responsive elements, and all members contained this element, indicating that the *MsbHLH* gene exhibited high responsiveness to light.

### 2.7. GO Enrichment Analysis of MsbHLHs

To further investigate the biological functions of *MsbHLH* genes in *M. sieboldii*, GO annotation (Table S7) and enrichment analysis (Figure S2) were performed on 142 *MsbHLH* genes in this study. *MsbHLH* genes were enriched for a total of 16 molecular functions, 7 cellular components, and 112 biological processes. Among the molecular functions, it was mainly enriched in processes such as transcriptional regulatory activity, DNA-binding transcription factor activity, and small molecule binding. Among the cellular components, they were mainly enriched in nucleus, intracellular membrane-bound organelles, and intracellular anatomical structures. Of the 82 genes enriched for biological processes, all are involved in processes such as RNA biosynthesis or metabolic regulation, cellular biosynthesis or metabolism, and gene expression. The rest were enriched in processes such as active regulation of biometabolic processes, regulation of response to red or far-red light, and photomorphogenesis.

### 2.8. Expression Pattern of MsbHLHs in Seeds

**Figure 9:**
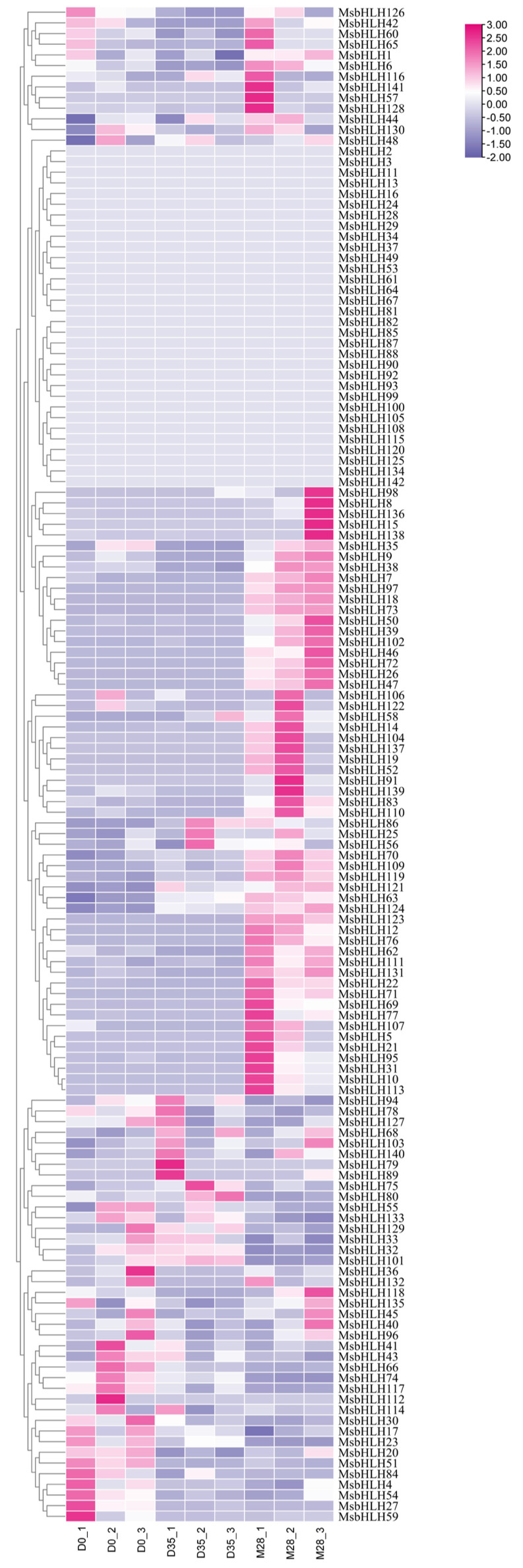
Expression profiles of *MsbHLHs* genes in different germination stages of *M. sieboldii* seeds.

To explore the possible functions of *MsbHLHs* during dormancy release and germination in *M. sieboldii*, we used RNA-seq data to characterize its expression after seed imbibition, stratification, and germination (Figure 9). A total of 49 gene family members showed a gradual increase in expression during seed release from dormancy to germination, 17 gene family members showed a gradual decrease in expression, and 32 genes were not expressed, mostly concentrated in subfamily 15. The bHLH homologous proteins in Arabidopsis were used as a reference, and the highest scoring ones were selected after BLAST to view their functions. MsbHLH3, MsbHLH37, MsbHLH81, MsbHLH90, and MsbHLH124 of subfamily 15 were found to be phytochrome-interacting factors that function in the phytochrome signaling pathway. Therefore, they were renamed *MsPIF4* (*MsbHLH3*), *MsPIF1* (*MsbHLH37*), *MsPIF7* (*MsbHLH81*), *MsPIF3a* (*MsbHLH90*), and *MsPIF3b* (*MsbHLH124*). Only the expression of *MsPIF3b* increased gradually, while the rest did not change perhaps because differential expression did not reach 2-fold. Since they all contain light-responsive elements in their promoters, it is hypothesized that there might be significant changes under different light quality stimuli.

To understand the potential roles of MsPIFs in different light-quality responses, we analyzed their expression using qRT-PCR. Prior to the light treatment of *M. sieboldii* seeds, we subjected it to 48 h of imbibition followed by 14 d of stratification. During this process, the expression of all *MsPIFs* increased after 48 h of imbibition, followed by a gradual decrease (Figure S3).

**Figure 10:**
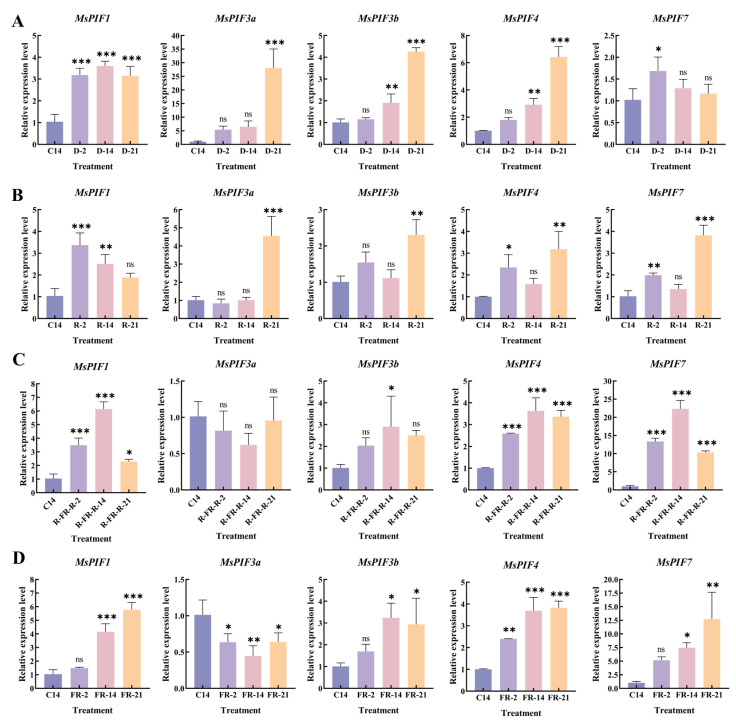
qRT-PCR analysis of *MsPIFs* under different light-quality treatments: (**A**) Under dark conditions. “C” indicates stratification. “D” indicates dark. Numbers indicate the number of days of treatment, same as below. (**B**) Under red light conditions. “R” indicates red light, same as below. (**C**) Under red-far-red-red light conditions. FR indicates far-red light, same as below. (**D**) Under far-red light conditions. Error lines indicate standard deviation. “ns” indicates no significant difference. Asterisks indicate significant differences in transcript levels compared with 14 d of stratification. (* *p* < 0.05, ** *p* < 0.01, *** *p* < 0.001).

After stratification, light treatments were performed. The results (Figure 10) showed that the expression of *MsPIF1*, *MsPIF3b*, *MsPIF4*, and *MsPIF7* gradually decreased under red light conditions in the pre-light period and seeds started to germinate (Figure S4). The opposite occurred in far-red light. It indicated that far-red light could positively regulate their expression changes and inhibit seed germination. Red light negatively regulated their expression and promoted seed germination. *MsPIF3a* expression was opposite to them in these two light qualities. Under the R-FR-R treatment, the trend of their expression changes was the same as that of far-red light. This suggests that far-red light counteracts the promotion of seed germination by red light. In the dark, *MsPIF1*, *MsPIF3a*, *MsPIF3b*, and *MsPIF4* expressions were increased, while *MsPIF7* expression was the opposite to them.

### 2.9. Subcellular Localization of MsPIFs

Full-length or UTR primers were designed for *MsPIF1*, *MsPIF3a*, *MsPIF3b*, *MsPIF4*, and *MsPIF7* gene sequences and amplified, and *MsPIFs* genes were successfully obtained (Figure S5).

**Figure 11:**
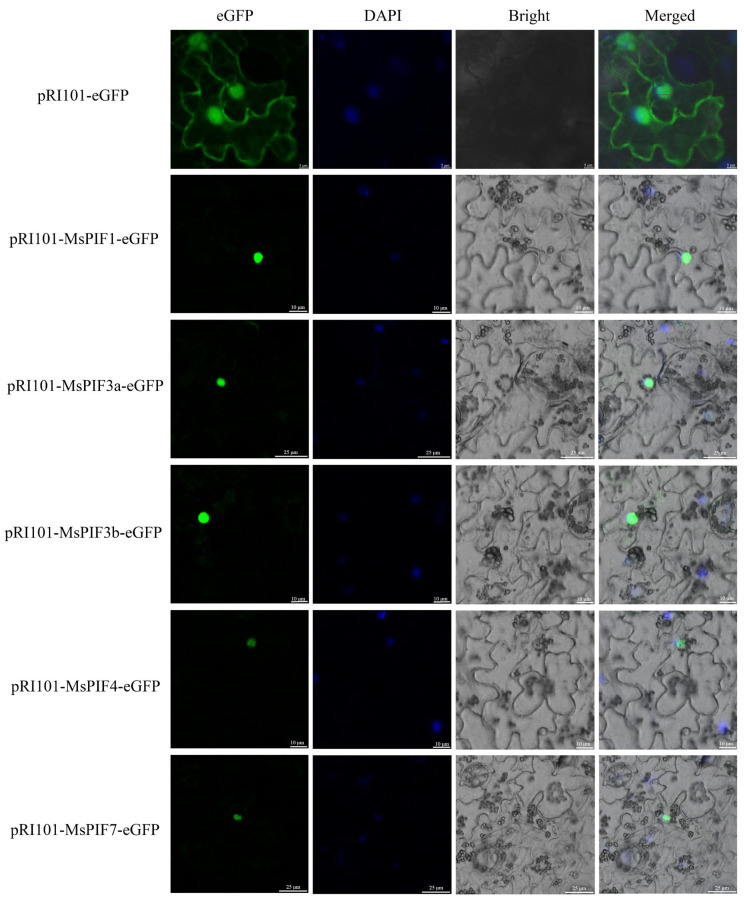
Subcellular localization of MsPIFs in tobacco leaves, where pRI101-eGFP is an empty control.

The subcellular localization of MsPIFs was determined by transient expression of the corresponding coding sequence fused to eGFP, and the location of the nucleus was confirmed using DAPI staining. As shown in Figure 11, all pRI101-MsPIFs-eGFP proteins were localized in the nucleus, which is consistent with previous subcellular prediction results. This suggests that MsPIFs play a biological role in the nucleus.

## 3. Discussion

bHLH TFs are present in all eukaryotes and are one of the largest families of transcription factors found in plants. Over the past two decades, bHLH TFs have been identified and characterized in a wide range of plant species, such as rice (183 *OsbHLHs*) [30], maize (231 *ZmbHLHs*) [30], rye (220 *ScbHLHs*) [31], and barley (103 *HvbHLHs*) [32] belonging to the monocots in angiosperms, and kale-type oilseed rape (602 *BnabHLHs*) [33], tomato (159 *SlbHLHs*) [34], Arabidopsis thaliana (162 *AtbHLHs*) [35], sweet orange (135 *CsbHLHs*) [36], passion fruit (138 *PebHLHs*) [28], Mongolian oak (89 *QmbHLHs*) [37], etc. The number of *bHLH* genes varies among species. Although *bHLH* has been identified in several species, the identification and characterization of *bHLH* in magnolias, which are located in the key position of evolutionary classification, have not yet been reported.

Not only does *M. sieboldii* belong to the magnolia group of plants, but its seeds also have morphophysiological dormancy properties [3]. In this study, we identified 142 genes encoding bHLH transcription factors of *M. sieboldii*. Among the 142 MsbHLHs, only MsbHLH13, MsbHLH16, MsbHLH19, and MsbHLH88 had an instability index of less than 40, which were 36.31, 36.07, 37.78, and 37.16, respectively. It was concluded that these four proteins with an instability index of less than 40 could exist stably. The overall folding structure of the proteins is hydrophilic groups outward and hydrophobic groups inward, and it forms a region of high hydrophobic value in the transmembrane region, according to which the position of secondary structures such as transmembrane helices of proteins can be determined. Except for MsbHLH138, the hydrophilicity scores of the remaining MsbHLH proteins were less than zero, indicating that they are all hydrophilic proteins. The results of subcellular localization prediction showed that MsbHLH was mainly localized in the nucleus, but some MsbHLH was also localized in other organelles. It was hypothesized that MsbHLH proteins might exercise different functions in different plant organelles, and these results laid the foundation for further studies on the biological functions of MsbHLH proteins.

Various studies have shown that the *bHLH* gene families in different plants are categorized into different numbers of subfamilies. The 218 *bHLH* genes in quinoa were categorized into 20 subfamilies and 1 ungrouped member [38]. The 128 *bHLH* members of birch were categorized into 21 subfamilies [39]. All 85 *bHLH* genes in ginkgo were categorized into 17 subfamilies [40], 159 *bHLH* members in tomato were categorized into 21 subfamilies [34], and 213 *bHLH* members in melon were categorized into 16 subfamilies and 4 orphan genes [41]. In this study, we classified 142 *MsbHLH* members into 27 subfamilies, and compared with Arabidopsis, *MsbHLH* genes contracted in 13 subfamilies (including subfamilies 1, 2, 4, 5, 6, 7, 8, 11, 15, 16, 18, 19, and 20) and expanded in 8 subfamilies (including subfamilies 9, 10, 13, 14, 17, 21, 23, 24).

Motif and gene structure analysis showed that members of the same subfamily of *MsbHLHs* tended to have similar motifs and gene structures. Motif 1 and motif 2 were present in the vast majority of MsbHLH proteins, which is consistent with the results obtained in Chinese white pear [42], tung tree [43], and cereal [44]. It indicates that MsbHLH transcription factors are relatively more conserved during plant evolution. However, there also exist some subfamily members that do not have motif 1 and motif 2 (e.g., MsbHLH85 and MsbHLH113 in subfamily 2 and MsbHLH103 in subfamily 20) or only have motif 1 or motif 2, suggesting that these MsbHLH proteins are not conserved during evolution. The evolution among them may have occurred at a much earlier stage, with the differentiation being more complex. Exon–intron as a structural differentiation, has an important role in the evolution of many gene families [45]. In this study, the exon–intron structure analysis of the genes showed that the number of exons–introns of *MsbHLH* genes was highly variable, ranging from 0 to 12, and 11 *MsbHLH* genes had no introns (*MsbHLH22*, *MsbHLH35*, *MsbHLH36*, *MsbHLH46*, *MsbHLH48*, *MsbHLH54*, *MsbHLH55*, *MsbHLH62*, *MsbHLH66*, *MsbHLH72*, and *MsbHLH89*), similar to grape [46], papaya [47], and jatropha [48]. *MsbHLH* genes in the same subfamily basically have similar gene structures [49], and exon–intron structure analysis of genes can provide insights into phylogenetic relationships [40].

Gene duplication events are essential for gene family expansion and genome evolution during plant evolution [50]. Chromosomal regions within 200 kb of two or more identical genomic regions are defined as tandem duplication events [51]. In the MsbHLH family of *M. sieboldii*, we identified a total of 4 tandem duplication pairs and 35 fragment duplicator pairs (Figure 6), suggesting that the role of fragment duplication is perhaps more important than tandem duplication. Selection pressure is expressed as Ka/Ks ratio values, with a Ka/Ks value of one indicating neutral evolution, Ka/Ks greater than one indicating positive selection (also known as Darwinian selection), and negative selection (as known as purifying selection) if Ka/Ks is less than one [42,52]. In all fragment duplication events in *MsbHLH*, all Ka/Ks values were less than one, indicating that the *MsbHLH* gene underwent purifying selection.

Analysis of *cis*-regulatory elements showed that the *cis*-acting elements of the MsbHLH promoters can be broadly categorized into three groups: elements related to stress response, hormone-responsive elements, and elements related to growth and development. Of these, the top four most numerous categories of *cis*-acting elements are the light-responsive element, abscisic acid-responsive element, MeJA-responsive element, and anaerobic induction, similar to passion fruit and plum [28,53]. The light-responsive elements were almost three times as numerous as the second most numerous. There are more of them than in *Passion fruit* [28] and *Phoebe bournei* [54]. The light-responsive element is a central molecular switch for biological adaptation to the light environment. It regulates development, metabolism, and stress response by integrating light signaling with gene expression networks. This was followed by abscisic and jasmonic acid-response elements. Abscisic acid plays a role in processes such as seed germination and plant response to abiotic stresses [55,56]. Abscisic acid and gibberellin induce and inhibit seed dormancy, respectively, and show opposite effects on seed germination [57,58,59]. MeJA is generally involved in stress tolerance, leaf senescence, and seed germination [60]. *AtPIFs* in Arabidopsis respond to the light environment through G-box elements [55]. *AtAIB* regulates drought-responsive genes through ABRE [61]. This suggests that some cis-acting elements are evolutionarily conserved in their core environmental response mechanisms. *MsbHLH* also has a higher number of gibberellin-responsive elements and auxin-responsive elements in the promoter region. It was shown that auxin would be involved in the regulation of jasmonic acid to abscisic acid signaling during seed germination in Arabidopsis [62]. These hormones crosstalk with each other during seed germination. Light also regulates seed dormancy and germination by modulating hormone metabolism and signaling pathways [63]. The diversity of cis-acting elements in the promoter region of *MsbHLHs* may be closely related to the different regulatory effects of *MsbHLHs* on the growth and development of *M. sieboldii* under different environmental conditions. Therefore, further functional studies of the *MsbHLH* genes could help to reveal their regulatory mechanisms on seed germination of *M. sieboldii* under different conditions.

Light is the most important environmental factor in forest ecosystems and one of the environmental resources with the most pronounced spatial and temporal variations [64]. Effective light within the forest is the key to determining whether many forest tree species can survive, grow, and complete regeneration in forest regeneration dynamics [65]. When solar radiation passes through the forest canopy, the ratio of red light to far-red light reaching the underside is altered to varying degrees, with red light weakening and far-red light enhancing [66]. It has been shown that bHLH family members have regulatory roles in seed germination and light signaling [14,15,67,68,69,70]. Among them, *MsbHLH3*, *MsbHLH37*, *MsbHLH81*, *MsbHLH90*, and *MsbHLH124* are all phytochrome interacting effectors after alignment to *Arabidopsis thaliana*. Previous studies claimed that phytochromes can interact with different groups of factors to regulate seed dormancy and germination, sheltering, phytohormone crosstalk, and clock-derived signaling pathways by integrating external environmental and internal signals [69,70]. In *Arabidopsis thaliana*, PIL5 (PIF3-like5 PIF1/bHLH15) is a germination inhibitor that decreases gibberellin levels in the dark, accelerates PIL5 degradation, and increases gibberellin bioactivity levels in the seed in the light [14]. AtPIF6 is strongly expressed during seed development and exists in two splice variants, and deletion of AtPIF6 increases primary seed dormancy [71]. AtPIF8 inhibits AtPHYA-mediated responses to far-red light, including seed germination. AtPIF8 protein levels are higher under far-red light irradiation than in darkness or red light, whereas other AtPIFs are higher in darkness than in red or far-red light [72]. AtPIF4 can physically interact with AtABI4 and act as a transcriptional complex to promote ABA biosynthesis and signaling. Eventually deepen primary seed dormancy [55]. In the present study, the expression of *MsPIFs* increased after the imbibition of *M. sieboldii* seeds. They gradually decreased during stratification, suggesting that the dormancy of *M. sieboldii* seeds may be gradually released during stratification. Subsequent light exposure of 10 h per day was carried out to simulate the average duration of daylight after seed abscission in the area where the samples were collected. The R treatment simulated a light environment with a large and stable R/FR within the forest, while the FR, on the contrary, and the R-FR-R treatments simulated light environments in which R/FR was in variation [73]. When under different light-quality treatments, only the seeds of the far-red light treatment did not germinate. Under this light quality, the expressions of *MsPIF1*, *MsPIF3b*, *MsPIF4*, and *MsPIF7* were all gradually elevated, contrary to those in darkness and red light where germination was higher. It tentatively suggests that they may play a role in inhibiting the germination of the *M. sieboldii* seeds under far-red light. *M. sieboldii* is a small tree, which has small seeds. When the seeds are shed, they tend to fall in the middle of the layer of dead branches and leaves. The far-red light content increases at this time, so the *M. sieboldii* seeds have difficulty germinating and the natural regeneration ability is weak. The expression of *MsPIF4* increased with the number of seed germinations under darkness and red light, which was hypothesized to be related to the gradual elongation of hypocotyls after seed germination (Figure S4). It has been shown that PIF4 is the main protein involved in the elongation of seedling hypocotyls [74,75,76].

## 4. Materials and Methods

### 4.1. Plant Materials and Light Quality Treatments

The seeds of *M. sieboldii* were collected from the Shenyang Botanical Garden. After imbibition for two days, they were mixed with wet sand that had been disinfected with potassium permanganate (KMnO_4_) [3] and subjected to low-temperature stratification at 4 °C for 14 days. Following this period, the seeds were separated from the wet sand and spread out in Petri dishes containing 1 cm of wet sand. They were then exposed to four different light treatments: dark (D) for 10 h, red light (R) for 10 h, far-red light (FR) for 10 h, and a combination of red light for 5 h, followed by far-red light for 2.5 h, and concluding with red light for an additional 2.5 h (R-FR-R). This is to simulate the light environment in the forest after seed shedding. Total light duration is the average winter day length in the simulated seed collection area. Three repetitions for each treatment. Samples were collected at various stages: dry seeds, after imbibition for 2 days, after stratification for 7 days, after stratification for 14 days, after 2 days of light exposure, after 14 days of light exposure, and after 21 days of light exposure. All collected samples were frozen with liquid nitrogen and stored at −80 °C for subsequent experiments.

### 4.2. Identification of bHLH Genes in the M. sieboldii Genome

The whole-genome sequence of *M. sieboldii* was obtained from previous laboratory work [77]. The sequences of all bHLH proteins in Arabidopsis were retrieved from the TAIR website (https://www.arabidopsis.org/, accessed on 30 March 2023) and subjected to BLAST comparison with the MsbHLH proteins of *M. sieboldii* to initially identify potential MsbHLH protein sequences. Based on the hidden Markov model of the bHLH domain (PF10010) obtained from the Pfam database (http://pfam.xfam.org/, accessed on 1 April 2023) [78], local HMMER (http://hmmer.janelia.org/, accessed on 3 April 2023) was employed to screen the seed model (*E*-value of 10^−5^) [79]. The MsbHLH protein sequences filtered by both methods were merged and deduplicated to preliminarily identify the MsbHLH family members. All predicted MsbHLH protein sequences were uploaded to CDD-Search for manual correction based on conserved domains, ultimately leading to the identification of the *MsbHLH* gene family members in *M. sieboldii*. The ExPASy ProtParam tool (https://web.expasy.org/protparam/, accessed on 7 April 2023) [80] was utilized to predict the physicochemical parameters of the proteins, including the number of amino acids, molecular weight, isoelectric point, instability index, grand average of hydrophilicity, and aliphatic index.

### 4.3. Phylogenetic Analysis of MsbHLHs

The protein sequences of all bHLH proteins from *Arabidopsis thaliana* were obtained from the TAIR website. Sequences lacking the bHLH domain were excluded, resulting in a total of 153 AtbHLH proteins being screened. The protein sequences of Arabidopsis AtbHLHs were aligned with the protein sequences of MsbHLHs using MUSCLE for multiple sequence alignment [81]. The phylogenetic tree was constructed using the Neighbor-Joining (NJ) method in MEGA-X software (Version 10.0.5) (bootstrap = 1000) [82], and the tree was visualized and enhanced using the online tool ITOL (https://itol.embl.de/tree/, accessed on 26 May 2023).

### 4.4. Gene Structure, Conserved Motif, and Cis-Regulatory Elements Analysis

The *MsbHLH* gene structure and intron–exon distributions were obtained using GFF annotation files from the *M. sieboldii*. The MEME online program (https://meme-suite.org/meme/tools/meme, accessed on 31 May 2023) [83] was employed to analyze the conserved motif in MsbHLH proteins, with the maximum number of motifs set to 10. Based on the genomic full-length DNA sequences of *MsbHLHs*, 2000 bp sequence upstream of each gene was retrieved by TBtools software (Version 2.200) using the PlantCare database (https://bioinformatics.psb.ugent.be/webtools/plantcare/html/, accessed on 18 June 2023) [84] to predict the *cis*-acting regulatory elements in the promoters of *MsbHLHs*. The results were then visualized using TBtools [85].

### 4.5. Duplication and Genome Synteny Analysis

All *MsbHLH* genes were anchored to the corresponding chromosomes based on the gene location information in the genome using Circos through the *M. sieboldii* genome file and the GFF annotation file.

The genomic data of *Arabidopsis thaliana*, poplar, tomato, blue star water lily, rice, and maize were downloaded from the EnsemblPlants database (http://plants.ensembl.org/index.html, accessed on 13 May 2023). The data were then analyzed for gene duplication events by One Step McScanX on TBtools and visualized using the Dual Synteny Plot.

The CDS sequences, protein sequences, and gene pair files of *MsbHLHs* were prepared and the Ka/Ks ratio was calculated using 1 as the cutoff value.

### 4.6. Protein–Protein Interaction Network Prediction, Homology Modeling, and GO Enrichment Analysis

All MsbHLH protein sequences were submitted to the STRING website (https://cn.string-db.org/, accessed on 1 September 2023). *Arabidopsis* homologous proteins were selected as references. The highest score proteins were used to construct the network after the BLAST step. Proteins that did not interact with other members were removed from the network. The secondary structures of MsbHLH proteins were predicted using the SOPMA server (https://npsa-pbil.ibcp.fr/, accessed on 8 November 2023) [86], and homology modeling to predict protein tertiary structure was performed using SWISS-MODEL (https://swissmodel.expasy.org/, accessed on 11 October 2023).

The Eggnog mapper (http://eggnog-mapper.embl.de/, accessed on 15 October 2023) [87] was employed for the purpose of gene function annotation, with the resulting data subsequently visualized via the Microbiology Letter website (https://www.bioinformatics.com.cn/, accessed on 11 October 2024).

### 4.7. Transcriptome and qRT-PCR Analysis

Transcriptome data were obtained from the pre-laboratory work [77], and TBtools software was employed to normalize FPKM values and visualize gene expression information.

The kit method (RNA Easy Fast Plant Tissue RNA Rapid Extraction Kit, TIANGEN, Beijing, China) was used to extract total RNA, while a reverse transcription kit (TransScript^®^ One-Step gDNA Removal and cDNA Synthesis SuperMix, TRAN, Beijing, China) was utilized for the reverse transcription assay. Real-time PCR was conducted using a quantification kit (SuperReal PreMix Plus (SYBR Green), TIANGEN, Beijing, China). The 60S was employed as an internal reference gene, with each gene undergoing three replicates. Gene alterations were calculated using the 2^−ΔΔCT^ method [88], and the data were analyzed with GraphPad Prism (Version 8.0.2). The primers utilized for qRT-PCR for each gene are presented in Table S8.

### 4.8. Subcellular Localization

*MsPIFs* were genetically cloned, and full-length or UTR primers (Table S8) were designed for *MsPIFs* gene sequences using the Primer Premier 5 and amplified using the PrimeSTAR HS enzyme (Takara, Beijing, China). The amplification products were gel-extracted (EasyPure Quick Gel Extraction Kit, TRAN, Beijing, China) and ligated with A-Tailing (Takara) and pMD18-T (Takara). The ligation products were transferred to *E. coli* DH5α (Takara), incubated at 37 °C for 12 h, and single clones were picked for PCR detection. The positive clones were sent to the Suzhou Jinwei Zhi Company (Suzhou, China) for sequencing.

Primers containing the homology arm sequence of the pRI101-eGFP vector (Table S8) were designed and the above positive plasmids were cloned into the pRI101-eGFP vector. The recombinant plasmids were transferred into tobacco leaves separately from the empty load. Three leaves were injected for each gene. After incubation in dark environment for 48 h and normal environment for 24 h, slides were made and observed on the machine (Laica, TCS SP8, Wetzlar, Germany).

## 5. Conclusions

In summary, 142 members of the *MsbHLH* gene family were identified from the genome of *M. sieboldii*, and they were classified into 27 subfamilies based on their phylogenetic relationship with Arabidopsis bHLH. Analysis of motif composition, gene structure, and homologous protein modeling indicated that the MsbHLH proteins of each subfamily were relatively conserved. But there were individual cases, which might be related to the unique evolutionary status of Magnoliaceae. Collinearity analysis suggested that the expansion of the *MsbHLH* gene family might have occurred primarily through segmental duplication. Protein interaction networks among MsbHLH may contribute to the diverse and complex regulatory networks among bHLH proteins. Promoter analysis showed that all MsbHLH members contained light-responsive elements, indicating that *MsbHLH* exhibits high light responsiveness. Most *MsbHLH* members functioned in the seed germination process. The expression of *MsPIF1*, *MsPIF4*, *MsPIF3b*, and *MsPIF7* gradually increased under far-red light, suggesting that they may inhibit seed germination in *M. sieboldii*. *M. sieboldii* faces difficulties in seed renewal under natural conditions. The above results identify the basis for further investigation of the regulatory role of bHLH proteins on seed dormancy and germination of *M. sieboldii* under different light qualities. Dissecting the *MsbHLH* gene involved in seed germination helps to optimize artificial breeding techniques. By taking the right measures, the development of seed embryos is accelerated and the breeding efficiency is improved. In turn, it effectively protects the endangered plant resources of the Magnolia genus and helps the recovery of natural populations.

## Data Availability

The original contributions presented in this study are included in the article and Supplementary Materials. Further inquiries can be directed to the corresponding author.

## Supplementary Materials

The supporting information can be downloaded at: https://www.mdpi.com/article/10.3390/ijms26073152/s1.
