# Supplementary material for: Genome-Wide Identification of the bHLH Gene Family in Magnolia sieboldii and Response of MsPIFs to Different Light Qualities
Source: Int J Mol Sci. 2025 Mar 28;26(7):3152. doi: 10.3390/ijms26073152 (PMC11989109; doi:10.3390/ijms26073152)
Supplement: Supplementary file 1 [file ijms-26-03152-s001.zip › ijms-3520030-supplementary/Table S8.pdf]

Table S8. List of primer sequences used in this study.

Primer Name	Sequence (5' to 3')
MsPIF1-DL-F	GGAGCCCATTTAGGTCCAAGG
MsPIF1-DL-R	GGTGAAGGCTGAGGTAGTGTTGATA
MsPIF3a-DL-F	AGGAAGCCGACTAAGTGCCC
MsPIF3a-DL-R	CGGTCCGCGTAAGTTTTGTATT
MsPIF3b-DL-F	AAACAATAGATCACCCGACCGT
MsPIF3b-DL-R	TTCGCTTGGCACTCTTACCC
MsPIF4-DL-F	GCAGCGGTGTTGGTGGTAAA
MsPIF4-DL-R	GGCACTCAGATTCTTCGGCAT
MsPIF7-DL-F	ATCAAGCCACCCACCATAAG
MsPIF7-DL-R	CCAGGAGATTCAAAAGACGC
MsPIF1-F	CTCGTTTCGTTTTAGATTGACTTCC
MsPIF1-R	ACAGGTTAGCCGCATCGTAGT
MsPIF3a-F	ATGCCTTACTCTGAATTCCATC
MsPIF3a-R	TTAACTGAATGGGGCATGGATCC
MsPIF3b-F	ATGCCTTACTCCGCAATTTATCCCA
MsPIF3b-R	CTACGTGGATGTCCTGATGGATGCA
MsPIF4-F	ATGAATCACTGCGTTCCTGATT
MsPIF4-R	CTAACCCAGATTGCCATTTTGA
MsPIF7-F	ATGAGTCAGTGTGTTGTACCCAACT
MsPIF7-R	CTAGTCATGTGGTGCACTGTTGGCT
MsPIF1-eGFP-F	ATGCCCGTCGACCCCGGGATGGAAGATGACTCTTCCGTCCC
MsPIF1-eGFP-R	GGATCCGGTACCCCCGGGACCGGACTCGTGAGTTTCTGGA
MsPIF3a-eGFP-F	ATGCCCGTCGACCCCGGGATGCCTTACTCTGAATTCCATC
MsPIF3a-eGFP-R	GGATCCGGTACCCCCGGGACTGAATGGGGCATGGATC
MsPIF3b-eGFP-F	ATGCCCGTCGACCCCGGGATGCCTTACTCCGCAATTTATCCC
MsPIF3b-eGFP-R	GGATCCGGTACCCCCGGGCGTGGATGTCCTGATGGATGC
MsPIF4-eGFP-F	ATGCCCGTCGACCCCGGGATGAATCACTGCGTTCCTGATTGG
MsPIF4-eGFP-R	GGATCCGGTACCCCCGGGACCCAGATTGCCATTTTGAATATTG
MsPIF7-eGFP-F	CGGGGGTACCGGATCCATGAGTCAGTGTGTTGTACCC
MsPIF7-eGFP-R	CCATGAATTCGGATCCGTCATGTGGTGCACTGTTGG
