# Supplementary figures and images for: Genome-Wide Identification of the bHLH Gene Family in Magnolia sieboldii and Response of MsPIFs to Different Light Qualities
Source: Int J Mol Sci. 2025 Mar 28;26(7):3152. doi: 10.3390/ijms26073152 (PMC11989109; doi:10.3390/ijms26073152)

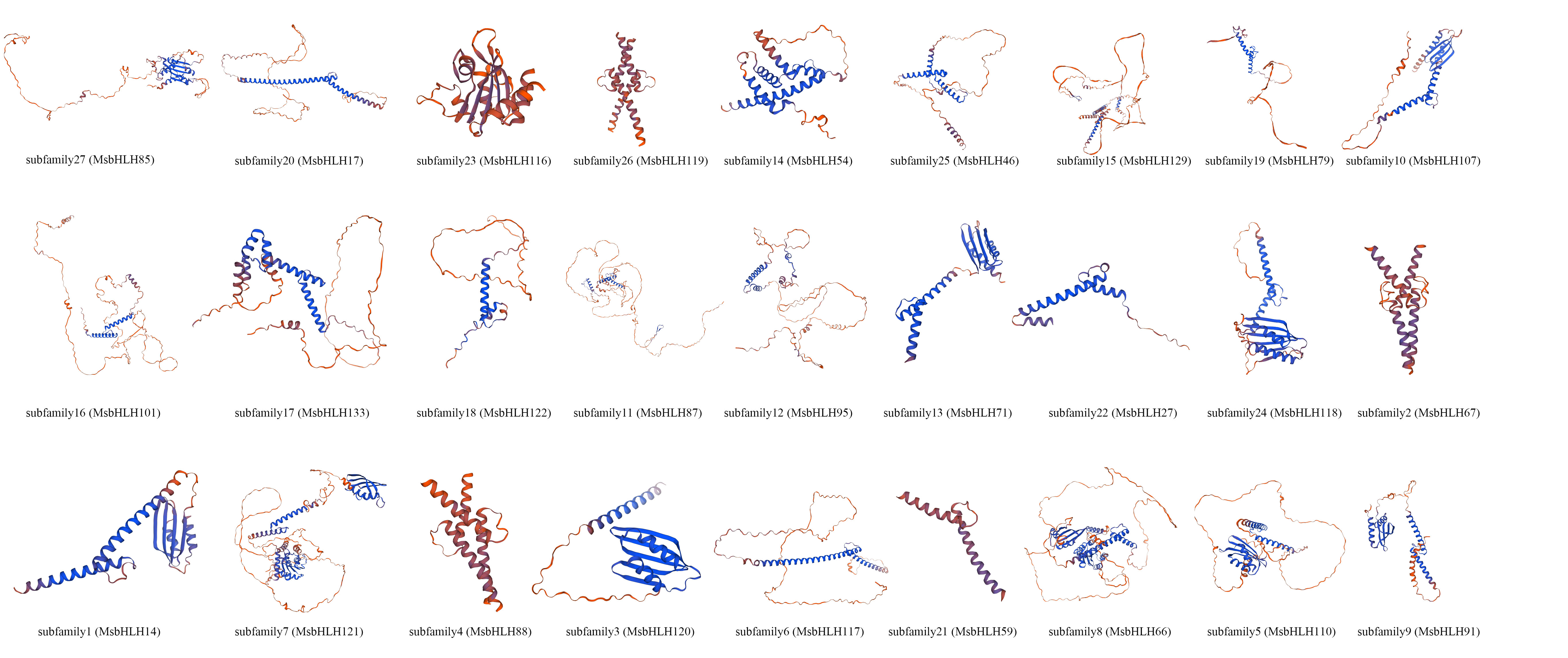

Supplement: Supplementary file 1 [file ijms-26-03152-s001.zip › ijms-3520030-supplementary/Figure S1.jpg]

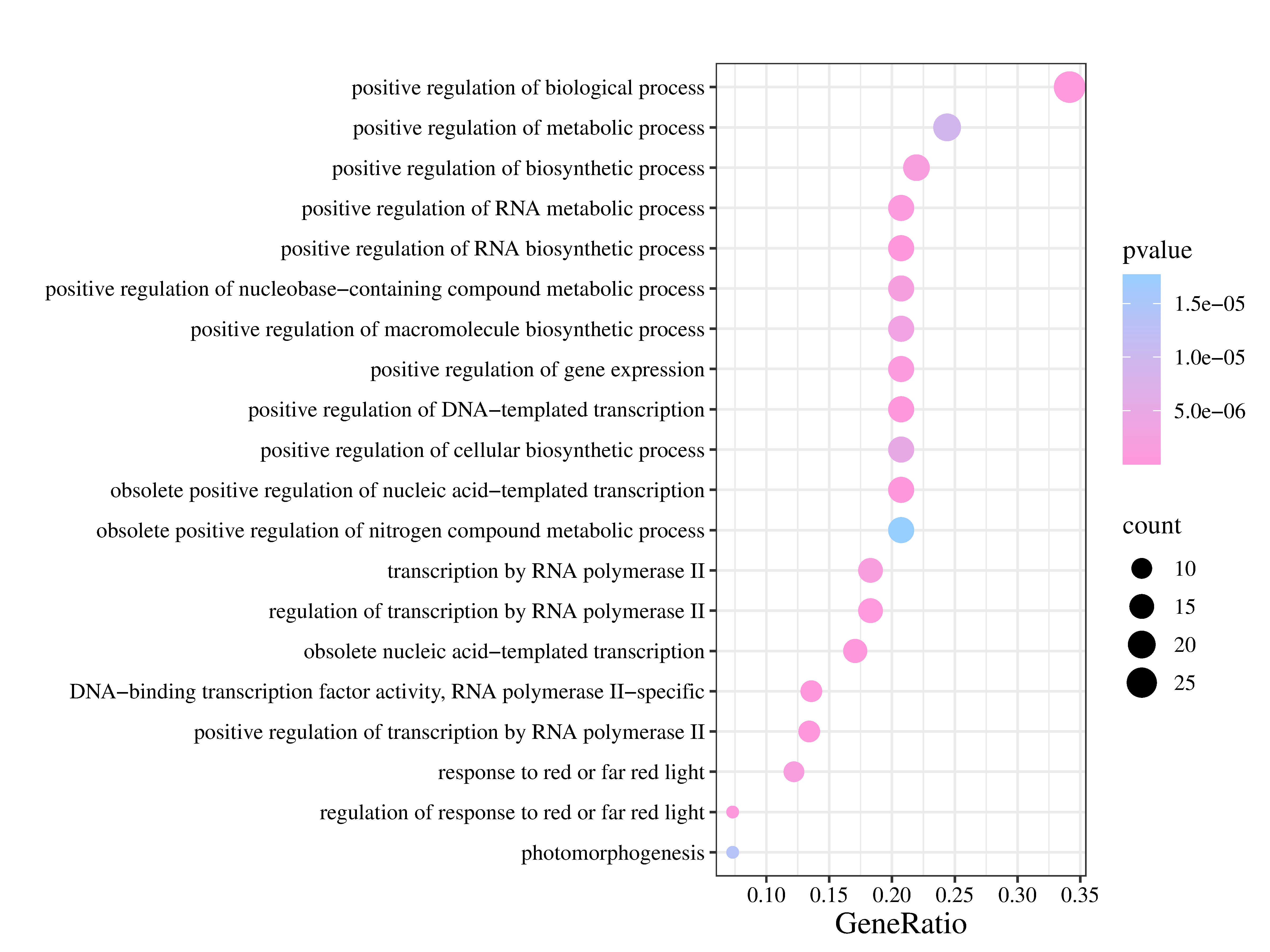

Supplement: Supplementary file 1 [file ijms-26-03152-s001.zip › ijms-3520030-supplementary/Figure S2.tiff]

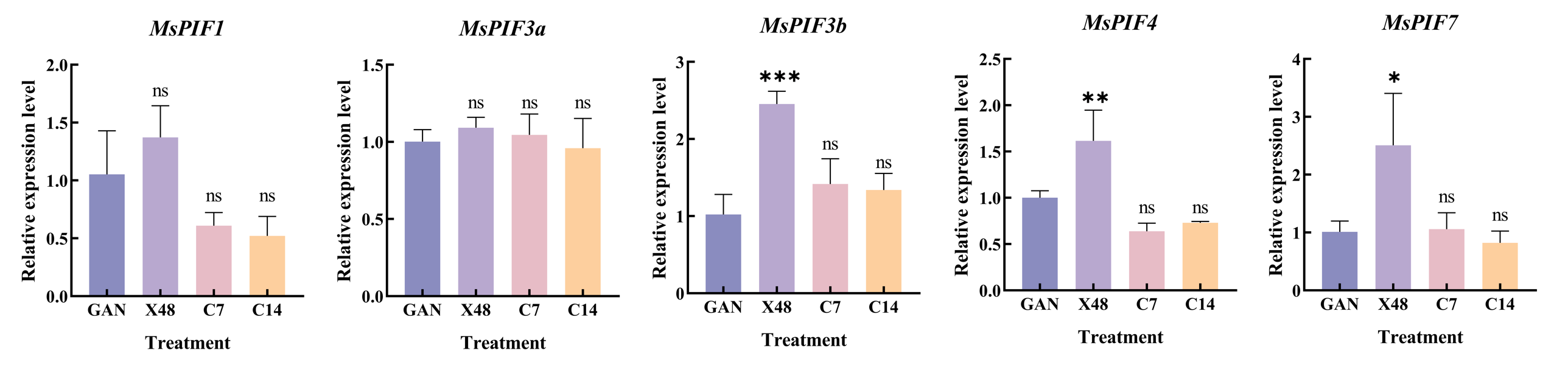

Supplement: Supplementary file 1 [file ijms-26-03152-s001.zip › ijms-3520030-supplementary/Figure S3.tif]

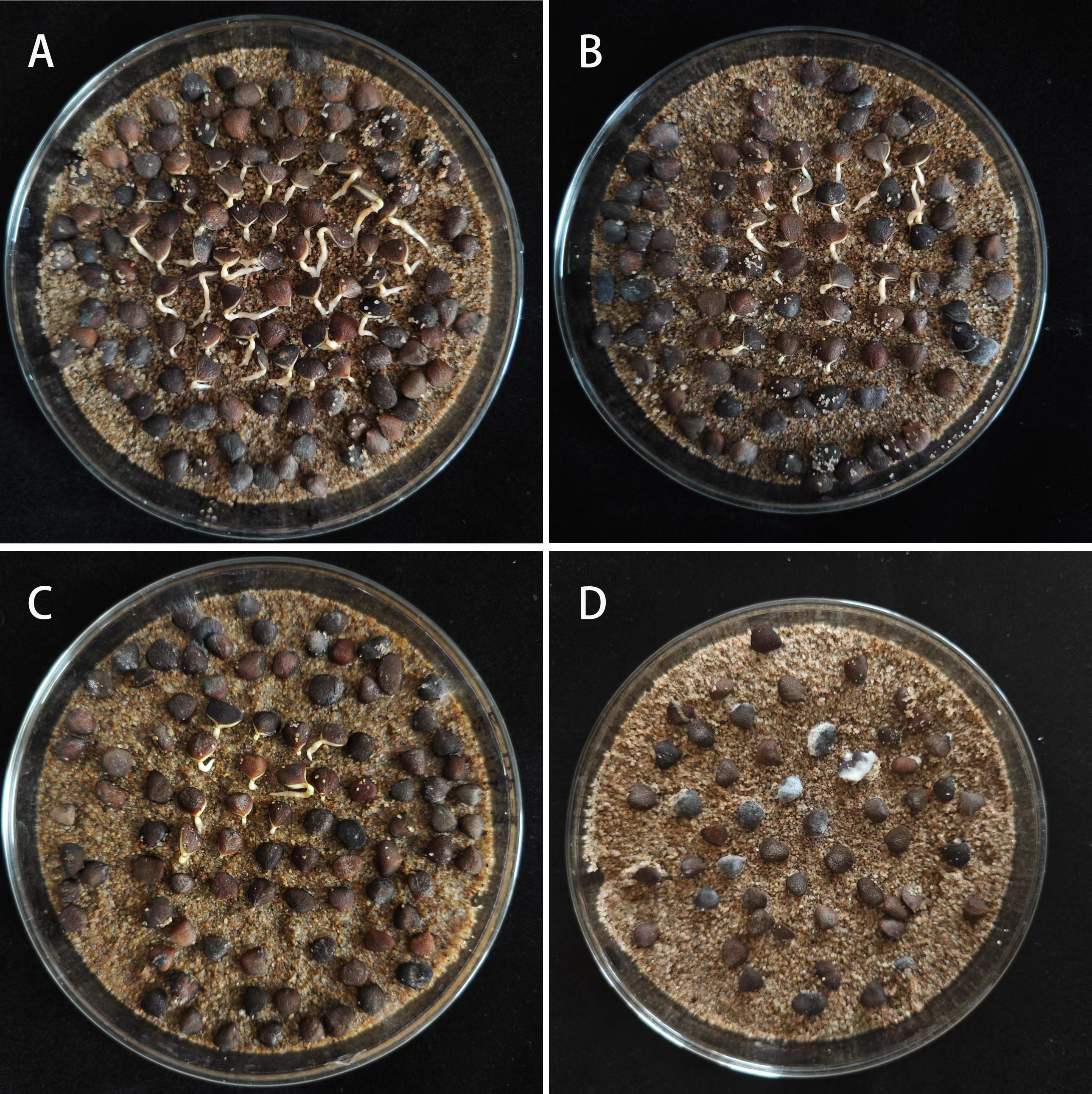

Supplement: Supplementary file 1 [file ijms-26-03152-s001.zip › ijms-3520030-supplementary/Figure S4.tif]

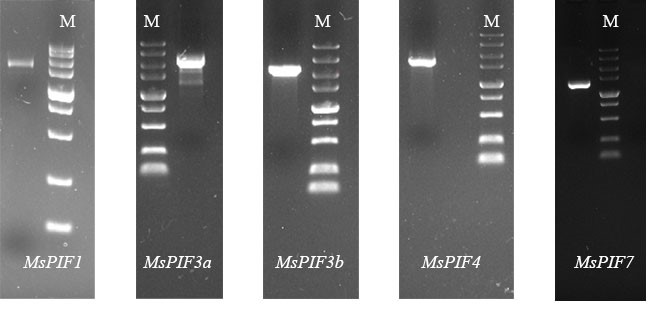

Supplement: Supplementary file 1 [file ijms-26-03152-s001.zip › ijms-3520030-supplementary/Figure S5.jpg]
